# Supplementary material for: Use of primary care and other healthcare services between age 85 and 90 years: longitudinal analysis of a single-year birth cohort, the Newcastle 85+ study
Source: BMJ Open. 2018 Jan 24;8(1):e019218. doi: 10.1136/bmjopen-2017-019218 (PMC5786130; doi:10.1136/bmjopen-2017-019218)
Supplement: Author's manuscript [file bmjopen-2017-019218.draft_revisions.pdf]

BMJ Open is committed to open peer review. As part of this commitment we make the peer review history of every article we publish publicly available.

When an article is published we post the peer reviewers' comments and the authors' responses online. We also post the versions of the paper that were used during peer review. These are the versions that the peer review comments apply to.

The versions of the paper that follow are the versions that were submitted during the peer review process. They are not the versions of record or the final published versions. They should not be cited or distributed as the published version of this manuscript.

BMJ Open is an open access journal and the full, final, typeset and author-corrected version of record of the manuscript is available on our site with no access controls, subscription charges or pay-per-view fees (http://bmjopen.bmj.com).

If you have any questions on BMJ Open's open peer review process please email editorial.bmjopen@bmj.com

BMJ Open

Use of primary care and other healthcare services by the very old; findings from the Newcastle 85+ Study.

Journal:	*BMJ Open*
Manuscript ID	bmjopen-2017-019218
Article Type:	Research
Date Submitted by the Author:	19-Aug-2017
Complete List of Authors:	Yadegarfar, Mohammad; Newcastle University, Institute of Health and Society/ Newcastle University Institute for Ageing (NUIA)<br>Jagger, Carol; University of Newcastle, Institute of Health and Society/ Newcastle University Institute for Ageing (NUIA)<br>Duncan, Rachel; Newcastle University, Institute of Health and Society/ Newcastle University Institute for Ageing (NUIA)<br>Fouweather, Tony; Newcastle University, Institute of Health and Society<br>Hanratty, Barbara; Newcastle University, Institute of Health and Society/ Newcastle University Institute for Ageing (NUIA)<br>Parker, Stuart; Newcastle University, Institute of Health and Society/ Newcastle University Institute for Ageing (NUIA)<br>Robinson, Louise; Newcastle University, Institute of Health and Society/ Newcastle University Institute for Ageing (NUIA)
**Primary Subject Heading**:	General practice / Family practice
Secondary Subject Heading:	Geriatric medicine, Health services research
Keywords:	HEALTH SERVICES ADMINISTRATION & MANAGEMENT, PRIMARY CARE, EPIDEMIOLOGY

SCHOLARONE™
Manuscripts

Use of primary care and other healthcare services by the very old; findings from the Newcastle 85+ Study.

Mohammad E. Yadegarfar [1,2], Carol Jagger [1,2], Rachel Duncan [1,2], Tony Fouweather [2], Barbara Hanratty [1,2], Stuart Parker [1,2] and Louise Robinson [1,2]

1. Newcastle University Institute for Ageing, Campus for Ageing and Vitality, Newcastle upon Tyne. NE4 5PL. United Kingdom.
2. Institute of Health and Society, Campus for Ageing and Vitality, Newcastle upon Tyne. NE4 5PL. United Kingdom.

Corresponding Author:

Professor Louise Robinson

Newcastle University Institute for Ageing

Campus for Ageing and Vitality

Newcastle upon Tyne

NE4 5PL

United Kingdom.

Email: a.l.robinson@ncl.ac.uk

Telephone: +44 (0) 191 208 7013

Key Words:

Health Services

Very Old

Primary Care

Newcastle 85+ Study

Word Count: 1950

For peer review only

For peer review only - http://bmjopen.bmj.com/site/about/guidelines.xhtml

Mohammad E. Yadegarfar, Institute of Health and Society, Campus for Ageing and Vitality, Newcastle upon Tyne. NE4 5PL. United Kingdom.
Research Assistant (Statistician)

Carol Jagger, Institute of Health and Society, Campus for Ageing and Vitality, Newcastle upon Tyne. NE4 5PL. United Kingdom.
AXA Professor of Epidemiology of Ageing

Rachel Duncan, Institute of Health and Society, Campus for Ageing and Vitality, Newcastle upon Tyne. NE4 5PL. United Kingdom.
Clinical Senior Lecturer in Ageing Research in Primary Care

Tony Fouweather, Newcastle University Institute for Ageing, Campus for Ageing and Vitality, Newcastle upon Tyne. NE4 5PL. United Kingdom.
Statistician

Barbara Hanratty, Institute of Health and Society, Campus for Ageing and Vitality, Newcastle upon Tyne. NE4 5PL. United Kingdom.
Professor of Primary Care & Public Health

Stuart Parker, Institute of Health and Society, Campus for Ageing and Vitality, Newcastle upon Tyne. NE4 5PL. United Kingdom.
Wm Leech Prof of Geriatric Medicine & CRN Specialty Cluster Lead

Louise Robinson, Institute of Health and Society, Campus for Ageing and Vitality, Newcastle upon Tyne. NE4 5PL. United Kingdom.
Professor of Primary Care and Ageing.

Louise Robinson is Guarantor for the paper.
Correspondence to: Louise Robinson: a.l.robinson@ncl.ac.uk

For peer review only

Abstract

Objective

To describe, using data from the Newcastle 85+ cohort study, the use of primary care services and other health care by 85 year olds as they age.

Design

Longitudinal population-based cohort study.

Setting

Newcastle upon Tyne and North Tyneside, United Kingdom.

Participants

Community-dwelling and institutionalised men and women recruited through general practices (n=845, 319 men and 526 women).

Results

Contact was established with 97% (n=1409/1459) of eligible 85 year olds, consent obtained from 74% (n=1042/1409) and 851 agreed to undergo the MDHA and a general practice medical records review. A total of 845 participants had complete data at baseline for this study (319 male, 526 female), with 344 (118 male, 226 female) re-interviewed at 60 months. After adjusting for confounders, all consultations significantly increased over the five years (IRR=1.03, 95%CI 1.01 to 1.05, p=0.001) as did GP consultations (IRR=1.03, 95%CI 1.01 to 1.05, p=0.006) but all primary care consultations decreased (IRR=0.96, 95%CI 0.94 to 0.98, p<0.001) as, by age 90, most primary care consultations were with the GP. Significant increases were also observed in inpatient and day hospital use over time though these disappeared after adjustment for confounders.

Conclusions

Our study of primary, secondary and community care use by the very old reveals that, between the ages of 85 and 90 years, older people are much more likely to consult their GP than other primary healthcare team members. With a rapidly ageing society, it is essential that GPs are appropriately skilled, and adequately supported by specialist colleagues, as the main healthcare provider for a population with complex and challenging needs.

For peer review only - http://bmjopen.bmj.com/site/about/guidelines.xhtml

Article Summary

Strengths and limitations of this study

- This study provided unique opportunity to analyse a large cohort of older adults' use of healthcare services extracted from GP medical records avoiding potential bias and inaccuracy emanating from self-reported or extracted research databases.
- Information on healthcare professional and consultation type provided much needed insight about the needs of this age group in both primary and secondary care settings.
- The absence of any information on consultation length and complexity precludes comment on the detailed nature of the increased workload in primary care.
- Our estimates of healthcare use are conservative, as consultations were analysed for 12 months prior to each interview and not the 12 months leading to death when healthcare use can be intensive.

For peer review only - http://bmjopen.bmj.com/site/about/guidelines.xhtml

Introduction

Our society is rapidly ageing. The fastest growing sector of our population is *the very old,* those aged 85 years and over; between 2015 and 2035, the older population of England and Wales (aged 65 years and over) is projected to increase by 48% whereas numbers aged 85 years and older will rise by 113% [1]. Findings from the first UK study to successfully recruit and retain a large cohort of people aged 85 and over [2] revealed multi-morbidity to be the norm [3], yet the majority remain able to live independently albeit with family support [3 4]. Alongside multi-morbidity, increasing age carries a greater risk physical frailty [5 6] and cognitive impairment and dementia [7]. Between 25-50% of those over 85 years are estimated to be frail [8], placing them at increased risk of death and disability and admission to hospital and long term care [9]. Dementia contributes a bigger disease burden than other long term illness such as cancer or stroke, with considerable care costs, especially in the last year of life [7 10].

Primary care services are central to the provision of health care in many developed countries, including the UK. Family physicians, or General Practitioners (GPs), and their teams provide the first point of contact for patients, diagnose disease, monitor long term conditions and have a pivotal role in disease prevention. It has long been acknowledged that primary care-led healthcare systems deliver more efficient and equitable services [11], with healthier, more satisfied patients, for lower cost and with fewer inequalities in both health and access to care [12 13]. With a rapidly ageing population, the resulting larger proportion experiencing multi-morbidity, cognitive decline and frailty, could place considerable pressures on health and social care provision, especially primary and community care services, in a system where the former is the first and main source of health care. However in the UK, primary care services are already almost at 'saturation point' with substantial increases in consultation rates and consultation duration with the population as a whole [14].

The aim of this paper is to describe, using data from the Newcastle 85+ study, the use of primary and secondary care services by a cohort of the very old as they age over a 5 year period.

Methods

The Newcastle 85+ Study is a prospective observational longitudinal study of a 1921 birth cohort who turned 85 during 2006 [2 3]. Potential participants were recruited from GP registered patient lists in Newcastle upon Tyne and North Tyneside: contact was established

For peer review only - http://bmjopen.bmj.com/site/about/guidelines.xhtml

with 97% (n=1409/1459) of eligible 85 year olds. Consent was obtained from 74% (n=1042/1409); 851 agreed to undergo detailed multidimensional health assessment (MDHA) and a general practice medical records review (GPRR); 3 consented to MDHA only; 188 consented to GPRR only and 358 declined all participation. Analysis of response, attrition and comparison with the national birth cohort have already been published [2 3].

As part of their GPRR, participant's primary health care use was recorded for the 12 months prior to their assessment interview (baseline, 36 and 60 months). Information gathered included consultations with 16 different professionals seen during these periods. Data for each participant was summarised in 3 ways: total number of consultations with each of the professionals separately; total number of consultations with any primary care professional (GP, GP out of hours, practice nurse/practitioner/HCA, community nurse, health visitor); and total number of visits to any of the 16 professionals (Table 1).

Additional information on secondary care use was collected for all participants at interview: inpatient, day hospital (total number of days spent in the 12 months prior to interview); outpatient and accidents and emergency (total number of visits in the 3 months prior to interview) (Table 1). Sociodemographic and health characteristics of participants were collected at baseline, 36 and 60 months follow up.

Statistical Analysis

Baseline sociodemographic (living status; self-rated health; education) and health characteristics (Mini-Mental State Examination (MMSE); Geriatric Depression score (GDS); disability; disease group count) of participants and sex differences were analysed using X^2 test for categorical data and Mann-Whitney U for count data. Trends in health care use over time were analysed by negative binomial regression as the data was over dispersed (variance much greater than mean). Zero-inflated negative binomial regression was used for outcomes where there was high numbers of zero consultations. Final models were adjusted for sex, sociodemographic and health characteristics. Confounding factors were measured at multiple time points (apart from education) and values were updated in models. Time trends were reported as incidence rate ratios (IRR). Primary and secondary care usage were analysed in the overall sample and in participants who took part at all three time points (baseline, 36, 60 months). All analyses were undertaken in Stata 12.0 (StataCorp; College Station, TX, USA).

For peer review only - http://bmjopen.bmj.com/site/about/guidelines.xhtml

Results

At baseline the study comprised 845 participants (319 men and 526 women) of whom 10.2% (n=86) were living in residential care, 12.5% (n=105) had moderate or severe cognitive impairment (MMSE score 18 or less), 6.3% (n=53) had severe disability and 18.6% (n=157) had four or more diseases (Table 2).

Between ages 85 and 90 years, the mean number of all consultations increased significantly by 2.9 extra consultations (p<0.001) and the mean number of GP consultations by 1.6 consultations (p<0.001) (Table 3). Nevertheless the increase in primary care consultations was not linear over the five year period, with an increase of 0.8 consultations between ages 85 and 87.5 years followed by a decrease of 3.1 consultations between ages 87.5 and 90 years, the latter due to a reduction in the mean number of consultations of any primary care professional apart from the GP. Indeed by age 90 primary care consultations were solely with the GP (Table 3). The same pattern of consultation use over time was found when the analysis was confined to participants who were alive at all three time points (Table 3). After adjustment for confounding factors there was a significant increase over the five years in all consultations (IRR=1.03, 95%CI 1.01 to 1.05, p=0.001) and GP consultations (IRR=1.03, 95%CI 1.01 to 1.05, p=0.006) but a significant decrease in all primary care consultations (IRR=0.96, 95%CI 0.94 to 0.98, p<0.001), and consultations with a community nurse (IRR=0.86, 95%CI 0.77 to 0.97, p=0.016) (Figure 1).

Analysis of the change in secondary care use between ages 85 and 90 years revealed a non-significant increase in mean inpatient days of 3.8 days (p=0.071), although when restricted to participants who survived to age 90 the mean inpatient days increased by 5 days (p=0.010) (Table 3). No significant changes in mean number of days as a day patient, outpatient or visits to A&E were found (Table 3). After adjustment for confounding factors, no significant trends over time were found for any of the secondary health care use (inpatient days, day hospital, outpatient visits, A&E visits) (Figure 2). Conclusions remained unchanged when analysis was confined to participants who survived the five years (data not shown).

Discussion

Our study suggests that over the age of 85years, older people are increasingly likely to consult their GP, rather than other members of the primary healthcare team. By the age of 90years, most primary care consultations are with the GP. In contrast, no significant changes were found in the use of secondary care services, including A & E and outpatient clinics.

For peer review only - http://bmjopen.bmj.com/site/about/guidelines.xhtml

These patterns remained after adjustment for changing sociodemographic factors (including admission to care homes and health factors such as multi morbidity and declining cognitive function. These findings help to explain the increasing workload in UK primary care; if GPs are consulting with the growing and increasingly complex population of 85 year olds, who show no increase in use of secondary care services [14]

Strengths and limitations

This study analysed a unique dataset on a large cohort of older adults' use of services. The extraction of data direct from GP medical records is a key strength, as it avoids the potential bias and inaccuracies of data that are self-reported or extracted from research databases. The absence of any information on consultation length and complexity precludes comment on the detailed nature of the increased workload in primary care. As there were no consultations for out of hours services, practice nurse and community nurse at 60 months (age 90), these consultations could only be analysed at baseline and 36 months due to model convergence. Consultations were analysed for the 12 months prior to each interview therefore excluded data on those who had not been interviewed at that time, mostly due to death. Our estimates of healthcare use are therefore conservative since healthcare use at end of life can be intensive in the 12 months leading to death.

In a majority of high income countries, general or family practice is the mainstay of health care, providing first line contacts and acting as gatekeeper to secondary care [13]. Our findings add further weight to the growing concern that NHS primary care will struggle to meet the needs of a rapidly ageing population, in the face of declining GP recruitment [15 16]. Recent research, looking at over 100 million primary care consultations for all age groups between 2007 and 2014, found that GP workload rose by more than 16% compared to <1% for practice nurses[14]; consultations rates were highest for the very young (< 4 years) and the very old (85 years). The authors concluded such an increase was probably an under-estimate, as the data excluded other GP duties such as administration and teaching. They also found that GP consultations were becoming longer. In England, an average GP consultation is 10 minutes, but longer for people aged over 65 years [17]. For people aged 85 and over where there are high rates of sensory impairment [3] [5] and multi-morbidity is the norm, such consultations may be longer and more complex. The skills required may explain the importance of the GP as healthcare provider to this population, despite the rapidly increasing role of nurses and nurse practitioners in primary care [18].

For peer review only - http://bmjopen.bmj.com/site/about/guidelines.xhtml

The number of nursing and residential home is decreasing [19], while the number of older people with significant care needs living at home is increasing [20]. This combination can only increase the pressure on primary and community care services [17 18], while continued financial austerity requires increased cost efficiency in service provision. Better access to geriatric expertise, through community-based multi-disciplinary assessment teams, may in future be beneficial to both patients and our primary gatekeeper healthcare services by providing the latter with easier access to specialist knowledge and support [17 18]. Although our findings currently reveal the GP as the key care provider for the very old, the crisis in recruitment of doctors suggest that the potential of specialist nurse practitioners to improve patient and care outcomes should be considered. Whether such a service would be acceptable to older people as an alternative to seeing the GP requires further exploration, but the integration of specialist palliative care nurses into routine NHS care provide an encouraging precedent. [21 22].

Finally, and most importantly, if GPs are to remain as the central care provider for our older people, they must be knowledgeable, skilled, and better supported by specialist colleagues and a clinical workforce which is trained and equipped to meet the needs and demands of a 21st century ageing population. It is interesting to note that in the UK, national recommendations to extend core GP training from 3 years to 4 years, with a focus on the management of age-related issues such as multi-morbidity, frailty and cognitive impairment and dementia, remain as recommendations and have not been translated into practice [23]. Future research is required to explore how best to configure services to address the health care needs of older people whilst maintaining quality of care.

For peer review only - http://bmjopen.bmj.com/site/about/guidelines.xhtml

References

1. Office for National Statistics ONS. National Population Projections: 2014-based Statistical Bulletin, 2015.
2. Davies K, Kingston A, Robinson L, et al. Improving retention of very old participants in longitudinal research: experiences from the Newcastle 85+ study. PloS one 2014;**9**(10):e108370 doi: 10.1371/journal.pone.0108370[published Online First: Epub Date]|.
3. Collerton J, Davies K, Jagger C, et al. Health and disease in 85 year olds: baseline findings from the Newcastle 85+ cohort study. Bmj 2009;**339**:b4904 doi: 10.1136/bmj.b4904[published Online First: Epub Date]|.
4. Jagger C, Collerton JC, Davies K, et al. Capability and dependency in the Newcastle 85+ cohort study. Projections of future care needs. BMC geriatrics 2011;**11**:21 doi: 10.1186/1471-2318-11-21[published Online First: Epub Date]|.
5. Clegg A, Young J, Iliffe S, et al. Frailty in elderly people. Lancet 2013;**381**(9868):752-62 doi: 10.1016/S0140-6736(12)62167-9[published Online First: Epub Date]|.
6. Rockwood K, Song X, MacKnight C, et al. A global clinical measure of fitness and frailty in elderly people. CMAJ : Canadian Medical Association journal = journal de l'Association medicale canadienne 2005;**173**(5):489-95 doi: 10.1503/cmaj.050051[published Online First: Epub Date]|.
7. Alzheimer's Disease International. World Alzheimer Report 2015. London, 2015:87.
8. Fried LP, Tangen CM, Walston J, et al. Frailty in older adults: evidence for a phenotype. The journals of gerontology Series A, Biological sciences and medical sciences 2001;**56**(3):M146-56
9. Song X, Mitnitski A, Rockwood K. Prevalence and 10-year outcomes of frailty in older adults in relation to deficit accumulation. Journal of the American Geriatrics Society 2010;**58**(4):681-7 doi: 10.1111/j.1532-5415.2010.02764.x[published Online First: Epub Date]|.
10. Alzheimer's Society. Dementia UK: Update. London, 2014.
11. Starfield B, Shi L, Macinko J. Contribution of primary care to health systems and health. The Milbank quarterly 2005;**83**(3):457-502 doi: 10.1111/j.1468-0009.2005.00409.x[published Online First: Epub Date]|.
12. Starfield B. The future of primary care: refocusing the system. The New England journal of medicine 2008;**359**(20):2087, 91 doi: 10.1056/NEJMp0805763[published Online First: Epub Date]|.
13. Starfield B, Shi L, Grover A, et al. The effects of specialist supply on populations' health: assessing the evidence. Health affairs 2005;**Suppl Web Exclusives**:W5-97-W5-107 doi: 10.1377/hlthaff.w5.97[published Online First: Epub Date]|.
14. Hobbs FD, Bankhead C, Mukhtar T, et al. Clinical workload in UK primary care: a retrospective analysis of 100 million consultations in England, 2007-14. Lancet 2016;**387**(10035):2323-30 doi: 10.1016/S0140-6736(16)00620-6[published Online First: Epub Date]|.
15. Roland M, Everington S. Tackling the crisis in general practice. Bmj 2016;**352**:i942 doi: 10.1136/bmj.i942[published Online First: Epub Date]|.
16. Thompson M, Walter F. Increases in general practice workload in England. Lancet 2016;**387**(10035):2270-2 doi: 10.1016/S0140-6736(16)00743-1[published Online First: Epub Date]|.
17. Royal College of General Practitioners R. The 2022 GP: Compendium of evidence, 2013.
18. Robinson L. Present and future configuration of health and social care services to enhance robustness in older age. London, 2015.

For peer review only - http://bmjopen.bmj.com/site/about/guidelines.xhtml

19. Matthews FE, Bennett H, Wittenberg R, et al. Who Lives Where and Does It Matter? Changes in the Health Profiles of Older People Living in Long Term Care and the Community over Two Decades in a High Income Country. PloS one 2016;**11**(9):e0161705 doi: 10.1371/journal.pone.0161705[published Online First: Epub Date]|.
20. Jagger C. Educational Disparities in Adult Disability: Person, Place, Policies, and Family. American journal of public health 2017;**107**(7):1021-22 doi: 10.2105/AJPH.2017.303859[published Online First: Epub Date]|.
21. Beswick AD, Rees K, Dieppe P, et al. Complex interventions to improve physical function and maintain independent living in elderly people: a systematic review and meta-analysis. Lancet 2008;**371**(9614):725-35 doi: 10.1016/S0140-6736(08)60342-6[published Online First: Epub Date]|.
22. Low LF, Yap M, Brodaty H. A systematic review of different models of home and community care services for older persons. BMC health services research 2011;**11**:93 doi: 10.1186/1472-6963-11-93[published Online First: Epub Date]|.
23. Gerada CR, B.; Simon, C.;. Preparing The Future GP: The Case for Enhanced GP Training: Royal College of Physicians, RCGP, 2012.

For peer review only

For peer review only - http://bmjopen.bmj.com/site/about/guidelines.xhtml

Table 1: Description of outcomes and confounding factors included

Variable	Variable Description	Variable Type
**Primary health care use**		
GP Practice	This variable records all consultations participants had with a health care professional 12 months prior to each MDHA at each time point.	Outcome
GP Practice out of hours		
Practice Nurse/Practitioner/HCA		
Community Nurse		
Health Visitor		
Dietician		
Phlebotomist		
Other		
Not Specified		
Clerical		
Pharmacist/Pharmacy Technician		
Chiropodist/Podiatrist		
Physiotherapist		
Counsellor/Practice Counsellor		
Psychiatrist		
Mental Health Worker		
**Secondary health care use**	Time spent by participants for each different type of hospital admission.	Outcome
Inpatient	Days spent during the 12 month prior to MDHA	
Day Patient		
Outpatient	Number of visits during the 3 months prior to MDHA	
A&E		
**Time**	This is a continuous measure of time in years from the start of baseline interview to participant's death.	Covariate
**Living status***		
Alone in community	Participant's living arrangements at each MDHA	Covariate
Not alone in community		
Institutional living		
**Self-Rated Health**	Participant's perception of their general	Covariate

For peer review only - http://bmjopen.bmj.com/site/about/guidelines.xhtml

Excellent/Very Good	health on a five point scale recoded into 3 categories.	
Good		
Fair/Poor		
**MMSE***		
Normal (26-30)	Participant's categorised MMSE scores at each MDHA.	Covariate
Mild (22-25)		
Mod (18-21)		
Severe (0-17)		
**GDS***		
No depression	Categorised Geriatric depression score collected at each MDHA.	Covariate
Mild		
Severe		
MMSE<15		
**Categorised Disability***		
None	Categorised disability score based on activities of daily living (ADLs), collected at each MDHA.	Covariate
1 - 6		
7 - 12		
13 - 17		
**Disease Groups***	Categorised disease groups (max 8). 8 Disease groups were identified with each scored 1 if the Participant's had a GP diagnoses of said disease at each GPRR. Disease groups included: Arthritis, Cancer, Cardiac disease, Cerebrovascular disease, Diabetes mellitus, Hypertension, Respiratory disease and Cognitive Impairment.	Covariate
0		
–1		
–2 – 3		
4+		

MDHA (Multidimensional Health Assessment); GPRR (GP Record Review); MMSE (Mini-Mental Estate Examination); GDS (Geriatric Depression Score);

For peer review only

For peer review only - http://bmjopen.bmj.com/site/about/guidelines.xhtml

Table 2: Baseline sociodemographic and health characteristics of the 85+ study participants

	Males (319)	Females (526) %(N)	All (845)	P-value
**Living Status***				
Alone in community	39.5 (126)	64.0 (336)	54.7 (462)	
Not alone in community	54.2 (173)	23.4 (123)	35.1 (296)	<0.001
Institutional living	6.3 (20)	12.6 (66)	10.2 (86)	
**Self-rated health***				
Excellent/Very Good	43.9 (137)	37.7 (193)	40.1 (330)	
Good	36.5 (114)	38.3 (196)	37.6 (310)	0.152
Fair/Poor	19.6 (61)	24.0 (123)	22.3 (184)	
**Education**				
0-9 Years	62.3 (195)	65.7 (339)	64.4 (534)	
10-11 Years	24.6 (77)	21.7 (112)	22.8 (189)	0.576
12+ Years	13.1 (41)	12.6 (65)	12.8 (106)	
**MMSE***				
Normal (26-30)	71.9 (228)	71.1 (371)	71.4 (599)	
Mild (22-25)	18.3 (58)	14.8 (77)	16.1 (135)	0.113
Mod (18-21)	3.5 (11)	6.9 (36)	5.6 (47)	
Severe (0-17)	6.3 (20)	7.3 (38)	6.9 (58)	
**GDS***				
No depression	79.7 (247)	71.4 (360)	74.6 (607)	
Mild	9.0 (28)	13.9 (70)	12.0 (98)	0.066
Severe	6.8 (21)	8.5 (43)	7.9 (64)	
MMSE<15	4.5 (14)	6.2 (31)	5.5 (45)	
**Categorised Disability***				
None	31.6 (100)	16.3 (85)	22.1 (185)	
1 - 6	52.4 (166)	57.5 (300)	55.5 (466)	<0.001
7 - 12	11.7 (37)	18.8 (98)	16.1 (135)	
13 - 17	4.4 (14)	7.5 (39)	6.3 (53)	
**Disease Groups***				
0	6.6 (21)	4.2 (22)	5.1 (43)	
1	19.4 (62)	21.5 (113)	20.7 (175)	0.448
2 – 3	55.5 (177)	55.7 (293)	55.6 (470)	
4+	18.5 (59)	18.6 (98)	18.6 (157)	

*Data available at each time point
MMSE (Mini-Mental Estate Examination); GDS (Geriatric Depression Score);

For peer review only - http://bmjopen.bmj.com/site/about/guidelines.xhtml

Table 3: Mean number of consultations (healthcare use) at each time point of the study for all participants, by sex

**All Participants (N=845)**	Baseline (N=845)	36 Months (N=485) Mean (SD)	60 Months (N=344)	P-value
All Consultations	10.4 (7.7)	11.4 (8.3)	13.3 (13.6)	<0.001
Primary Care Consultations	9.8 (7.5)	10.6 (7.8)	7.5 (6.5)	0.026
GP	5.9 (4.8)	6.5 (5.9)	7.5 (6.5)	<0.001
GP out of hours service**	0.1 (0.5)	0.2 (0.8)	0.0 (0.0)	0.575
Practice Nurse/Practitioner/HCA**	2.8 (3.0)	2.6 (3.0)	0.0 (0.0)	0.634
Community Nurse**	1.0 (3.9)	1.1 (3.0)	0.0 (0.0)	0.823
Clerical	0.3 (0.7)	0.3 (1.6)	5.8 (10.7)	<0.001
Pharmacist/Pharmacy Technician	0.1 (0.3)	0.0 (0.0)	0.0 (0.3)	0.693
All Other Consults	0.2 (0.7)	0.5 (1.1)	0.0 (0.5)	<0.001
Inpatient	3.6 (15.3)	4.6 (14.0)	7.4 (18.6)	0.071
Day Patient	0.2 (0.9)	0.2 (0.6)	0.1 (0.4)	0.027
Outpatient***	0.6 (1.8)	0.6 (1.2)	0.6 (1.9)	0.974
A & E***	0.1 (0.3)	0.1 (0.4)	0.1 (0.4)	0.500
**Participants Alive at 60 Months (N=344)**	**(N=344)**	**(N=344)**	**(N=344)**	
All Consultations	9.9 (6.6)	10.8 (8.1)	13.3 (13.6)	<0.001
Primary Care Consultations	9.4 (6.5)	10.0 (7.5)	7.5 (6.5)	0.033
GP	5.7 (4.5)	6.2 (6.0)	7.5 (6.5)	<0.001
GP out of hours service**	0.1 (0.3)	0.2 (0.9)	0.0 (0.0)	<0.001
Practice Nurse/Practitioner/HCA**	3.2 (3.3)	2.8 (2.9)	0.0 (0.0)	0.161
Community Nurse**	0.5 (2.0)	0.8 (2.0)	0.0 (0.0)	0.473
Clerical	0.3 (0.7)	0.4 (1.8)	5.8 (10.7)	<0.001
Pharmacist/Pharmacy Technician	0.1 (0.3)	0.0 (0.2)	0.0 (0.3)	0.448
All Other Consults	0.2 (0.6)	0.4 (1.1)	0.0 (0.1)	<0.001
Inpatient	2.4 (9.9)	3.5 (11.5)	7.4 (18.6)	0.010
Day Patient	0.2 (0.7)	0.2 (0.6)	0.1 (0.4)	0.373
Outpatient (Last 3 Months)	0.5 (1.0)	0.5 (1.2)	0.6 (1.9)	0.069
A & E (Last 3 Months)	0.1 (0.3)	0.1 (0.4)	0.1 (0.4)	0.896

*p-value for change over time
** Analysis based on baseline and 36 months as there were no non-zero observations at 60 months
*** Numbers based on 3 months prior to interview

For peer review only - http://bmjopen.bmj.com/site/about/guidelines.xhtml

For peer review only

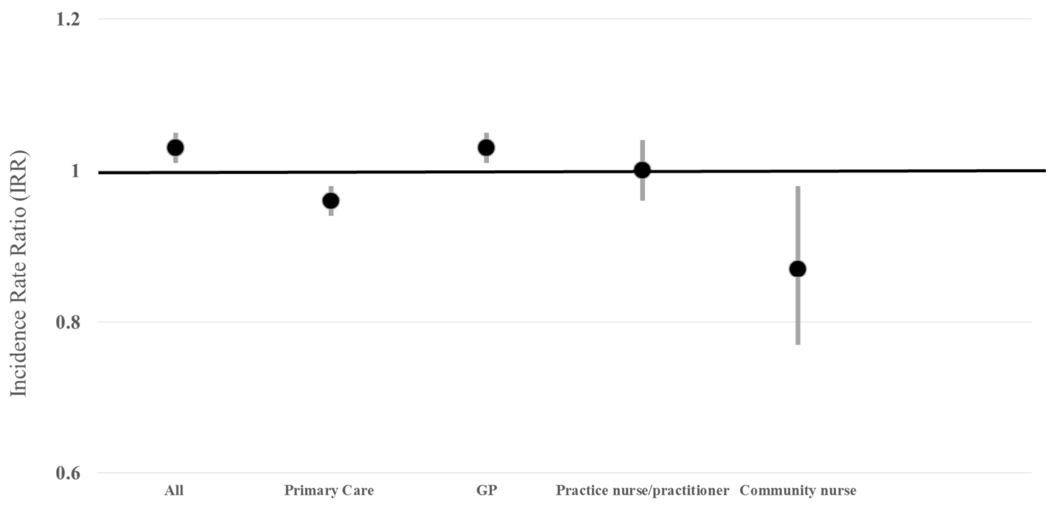

Figure 1: Time trends in primary and community care consultations (IRR and 95% CI) adjusted for sex, living status, self-rated health, MMSE (Mini-Mental Estate Examination), GDS (geriatric depression score) and disease groups count.

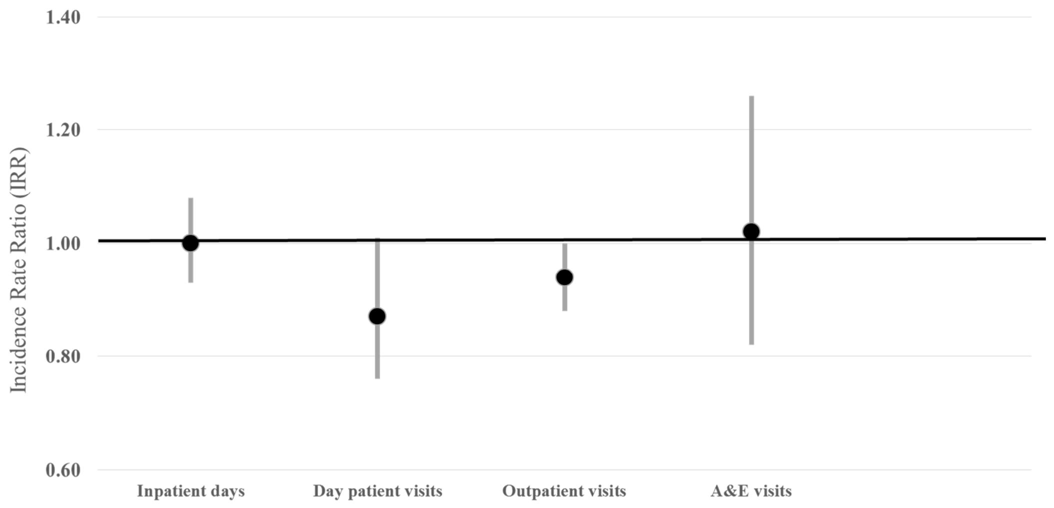

Figure 2: Time trends in secondary care consultations (IRR and 95% CI) adjusted for sex, living status, self-rated health, MMSE (Mini-Mental Estate Examination), GDS (geriatric depression score) and disease groups count.

For peer review only - http://bmjopen.bmj.com/site/about/guidelines.xhtml

Contributor statement

LR conceived the study and obtained project funding. MEY, CJ and TF were responsible for data analysis. All authors contributed to drafting of the paper and approved the final manuscript.

Funding

UK MRC and BBSRC (G0500997), Dunhill Medical Trust (R124/0509); Newcastle Healthcare Charity; NIHR Newcastle Biomedical Research Centre. The funding sources had no role in the study design; in the collection, analysis and interpretation of data; in the writing of the report, or in the decision to submit the paper for publication. This paper presents independent research funded by the National Institute for Health Research School for Primary Care Research (NIHR SPCR), project number SPCR 303. Louise Robinson is funded by a National Institute for Health Research Professorship (NIHR-RP-011-043). The views expressed are those of the author(s) and not necessarily those of the NIHR, the National Health Service or the Department of Health.

Acknowledgements

Thanks are especially due to the 85 year olds of Newcastle and North Tyneside, and their families and carers, for the generous donation of their time and personal information. In addition we thank the research nurses, data manager, project secretary, and the North of England Commissioning Support Unit and local general practitioners and their staff.

Copyright

The Corresponding Author has the right to grant on behalf of all authors and does grant on behalf of all authors, a worldwide licence to the Publishers and its licensees in perpetuity, in all forms, formats and media (whether known now or created in the future), to i) publish, reproduce, distribute, display and store the Contribution, ii) translate the Contribution into other languages, create adaptations, reprints, include within collections and create summaries, extracts and/or, abstracts of the Contribution, iii) create any other derivative work(s) based on the Contribution, iv) to exploit all subsidiary rights in the Contribution, v) the inclusion of electronic links from the Contribution to third party material where-ever it may be located; and, vi) licence any third party to do any or all of the above.

Competing interest statement

For peer review only - http://bmjopen.bmj.com/site/about/guidelines.xhtml

All authors have completed the Unified Competing Interest form and declared any relevant support from any organisation for the submitted work in the previous three years.

Transparency declaration

LR affirms that the manuscript is an honest, accurate, and transparent account of the study being reported; that no important aspects of the study have been omitted; and that any discrepancies from the study as planned (and, if relevant, registered) have been explained.

Data Sharing Statement

No additional data is available.

For peer review only

For peer review only - http://bmjopen.bmj.com/site/about/guidelines.xhtml

STROBE 2007 (v4) Statement—Checklist of items that should be included in reports of *cohort studies*

Section/Topic	Item #	Recommendation	Reported on page #
**Title and abstract**	1	(*a*) Indicate the study's design with a commonly used term in the title or the abstract	1 and 3
		(*b*) Provide in the abstract an informative and balanced summary of what was done and what was found	3
**Introduction**			
Background/rationale	2	Explain the scientific background and rationale for the investigation being reported	5
Objectives	3	State specific objectives, including any prespecified hypotheses	5
**Methods**			
Study design	4	Present key elements of study design early in the paper	5/6
Setting	5	Describe the setting, locations, and relevant dates, including periods of recruitment, exposure, follow-up, and data collection	5/6
Participants	6	(*a*) Give the eligibility criteria, and the sources and methods of selection of participants. Describe methods of follow-up	5/6
		(*b*) For matched studies, give matching criteria and number of exposed and unexposed	-
Variables	7	Clearly define all outcomes, exposures, predictors, potential confounders, and effect modifiers. Give diagnostic criteria, if applicable	6
Data sources/ measurement	8*	For each variable of interest, give sources of data and details of methods of assessment (measurement). Describe comparability of assessment methods if there is more than one group	*6*
Bias	9	Describe any efforts to address potential sources of bias	6
Study size	10	Explain how the study size was arrived at	6
Quantitative variables	11	Explain how quantitative variables were handled in the analyses. If applicable, describe which groupings were chosen and why	6
Statistical methods	12	(*a*) Describe all statistical methods, including those used to control for confounding	6
		(*b*) Describe any methods used to examine subgroups and interactions	6
		(*c*) Explain how missing data were addressed	6
		(*d*) If applicable, explain how loss to follow-up was addressed	6
		(*e*) Describe any sensitivity analyses	6
**Results**			

For peer review only

Participants	13*	(a) Report numbers of individuals at each stage of study—eg numbers potentially eligible, examined for eligibility, confirmed eligible, included in the study, completing follow-up, and analysed	6/7
		(b) Give reasons for non-participation at each stage	7
		(c) Consider use of a flow diagram	-
Descriptive data	14*	(a) Give characteristics of study participants (eg demographic, clinical, social) and information on exposures and potential confounders	7
		(b) Indicate number of participants with missing data for each variable of interest	-
		(c) Summarise follow-up time (eg, average and total amount)	7
Outcome data	15*	Report numbers of outcome events or summary measures over time	7
Main results	16	(*a*) Give unadjusted estimates and, if applicable, confounder-adjusted estimates and their precision (eg, 95% confidence interval). Make clear which confounders were adjusted for and why they were included	7
		(*b*) Report category boundaries when continuous variables were categorized	7
		(*c*) If relevant, consider translating estimates of relative risk into absolute risk for a meaningful time period	7
Other analyses	17	Report other analyses done—eg analyses of subgroups and interactions, and sensitivity analyses	7
**Discussion**			
Key results	18	Summarise key results with reference to study objectives	7
**Limitations**			
Interpretation	20	Give a cautious overall interpretation of results considering objectives, limitations, multiplicity of analyses, results from similar studies, and other relevant evidence	8
Generalisability	21	Discuss the generalisability (external validity) of the study results	8
**Other information**			
Funding	22	Give the source of funding and the role of the funders for the present study and, if applicable, for the original study on which the present article is based	9

*Give information separately for cases and controls in case-control studies and, if applicable, for exposed and unexposed groups in cohort and cross-sectional studies.

Note: An Explanation and Elaboration article discusses each checklist item and gives methodological background and published examples of transparent reporting. The STROBE checklist is best used in conjunction with this article (freely available on the Web sites of PLoS Medicine at http://www.plosmedicine.org/, Annals of Internal Medicine at http://www.annals.org/, and Epidemiology at http://www.epidem.com/). Information on the STROBE Initiative is available at www.strobe-statement.org.

For peer review only

BMJ Open

Use of primary care and other healthcare services between age 85 and 90: longitudinal analysis of a single year birth cohort, the Newcastle 85+ Study.

Journal:	*BMJ Open*
Manuscript ID	bmjopen-2017-019218.R1
Article Type:	Research
Date Submitted by the Author:	27-Oct-2017

Complete List of Authors:	Yadegarfar, Mohammad; Newcastle University, Institute of Health and Society/ Newcastle University Institute for Ageing (NUIA)<br>Jagger, Carol; University of Newcastle, Institute of Health and Society/ Newcastle University Institute for Ageing (NUIA)<br>Duncan, Rachel; Newcastle University, Institute of Health and Society/ Newcastle University Institute for Ageing (NUIA)<br>Fouweather, Tony; Newcastle University, Institute of Health and Society<br>Hanratty, Barbara; Newcastle University, Institute of Health and Society/ Newcastle University Institute for Ageing (NUIA)<br>Parker, Stuart; Newcastle University, Institute of Health and Society/ Newcastle University Institute for Ageing (NUIA)<br>Robinson, Louise; Newcastle University, Institute of Health and Society/ Newcastle University Institute for Ageing (NUIA)

**Primary Subject Heading**:	General practice / Family practice
Secondary Subject Heading:	Geriatric medicine, Health services research
Keywords:	HEALTH SERVICES ADMINISTRATION & MANAGEMENT, PRIMARY CARE, EPIDEMIOLOGY

SCHOLARONE™
Manuscripts

For peer review only - http://bmjopen.bmj.com/site/about/guidelines.xhtml

Use of primary care and other healthcare services between age 85 and 90: longitudinal analysis of a single year birth cohort, the Newcastle 85+ Study.

Mohammad E. Yadegarfar [1,2], Carol Jagger [1,2] , Rachel Duncan [1,2], Tony Fouweather [2], Barbara Hanratty [1,2], Stuart Parker [1,2] and Louise Robinson [1,2]

1. Newcastle University Institute for Ageing, Campus for Ageing and Vitality, Newcastle upon Tyne. NE4 5PL. United Kingdom.
2. Institute of Health and Society, Campus for Ageing and Vitality, Newcastle upon Tyne. NE4 5PL. United Kingdom.

Corresponding Author:

Professor Louise Robinson

Newcastle University Institute for Ageing

Campus for Ageing and Vitality

Newcastle upon Tyne

NE4 5PL

United Kingdom.

Email: a.l.robinson@ncl.ac.uk

Telephone: +44 (0) 191 208 7013

Key Words:

Health Services

Very Old

Primary Care

Newcastle 85+ Study

Word Count: 1950

For peer review only

Mohammad E. Yadegarfar, Institute of Health and Society, Campus for Ageing and Vitality, Newcastle upon Tyne. NE4 5PL. United Kingdom.

Research Assistant (Statistician)

Carol Jagger, Institute of Health and Society, Campus for Ageing and Vitality, Newcastle upon Tyne. NE4 5PL. United Kingdom.

AXA Professor of Epidemiology of Ageing

Rachel Duncan, Institute of Health and Society, Campus for Ageing and Vitality, Newcastle upon Tyne. NE4 5PL. United Kingdom.

Clinical Senior Lecturer in Ageing Research in Primary Care

Tony Fouweather, Newcastle University Institute for Ageing, Campus for Ageing and Vitality, Newcastle upon Tyne. NE4 5PL. United Kingdom.

Statistician

Barbara Hanratty, Institute of Health and Society, Campus for Ageing and Vitality, Newcastle upon Tyne. NE4 5PL. United Kingdom.

Professor of Primary Care & Public Health

Stuart Parker, Institute of Health and Society, Campus for Ageing and Vitality, Newcastle upon Tyne. NE4 5PL. United Kingdom.

Wm Leech Prof of Geriatric Medicine & CRN Specialty Cluster Lead

Louise Robinson, Institute of Health and Society, Campus for Ageing and Vitality, Newcastle upon Tyne. NE4 5PL. United Kingdom.

Professor of Primary Care and Ageing.

Louise Robinson is Guarantor for the paper.

Correspondence to: Louise Robinson: a.l.robinson@ncl.ac.uk

For peer review only

Abstract

Objective

To describe, using data from the Newcastle 85+ cohort study, the use of primary care services and other health care by 85 year olds as they age.

Design

Longitudinal population-based cohort study.

Setting

Newcastle upon Tyne and North Tyneside, United Kingdom.

Participants

Community-dwelling and institutionalised men and women recruited through general practices (n=845, 319 men and 526 women).

Results

Contact was established with 97% (n=1409/1459) of eligible 85 year olds, consent obtained from 74% (n=1042/1409) and 851 agreed to undergo the multidimensional health assessment and a general practice medical records review. A total of 845 participants had complete data at baseline for this study (319 male, 526 female), with 344 (118 male, 226 female) re-interviewed at 60 months. After adjusting for confounders, all consultations significantly increased over the five years (Incidence rate ratio, IRR=1.03, 95%CI 1.01 to 1.05, p=0.001) as did general practitioner (GP) consultations (IRR=1.03, 95%CI 1.01 to 1.05, p=0.006). Significant increases were also observed in inpatient and day hospital use over time though these disappeared after adjustment for confounders.

Conclusions

Our study of primary, secondary and community care use by the very old reveals that, between the ages of 85 and 90 years, older people are much more likely to consult their GP than any other primary healthcare team members. With a rapidly ageing society, it is essential that both current and future GPs are appropriately skilled, and adequately supported by specialist colleagues, as the main healthcare provider for a population with complex and challenging needs.

For peer review only - http://bmjopen.bmj.com/site/about/guidelines.xhtml

Article Summary

Strengths and limitations of this study

- This study provided unique opportunity to analyse a large cohort of older adults' use of healthcare services extracted from GP medical records avoiding potential bias and inaccuracy emanating from self-reported or extracted research databases.
- Information on healthcare professional and consultation type provided much needed insight about the needs of this age group in both primary and secondary care settings.
- The absence of any information on consultation length and complexity precludes comment on the detailed nature of the increased workload in primary care.
- Our estimates of healthcare use are conservative, as consultations were analysed for 12 months prior to each interview and not the 12 months leading to death when healthcare use can be intensive.

For peer review only

Introduction

Our society is rapidly ageing. The fastest growing sector of our population is *the very old,* those aged 85 years and over; between 2015 and 2035, the older population of England and Wales (aged 65 years and over) is projected to increase by 48% whereas numbers aged 85 years and older will rise by 113% [1]. Findings from the first UK study to successfully recruit and retain a large cohort of people aged 85 and over [2] revealed multi-morbidity to be the norm [3], yet the majority remain able to live independently albeit with family support [3, 4]. Alongside multi-morbidity, increasing age carries a greater risk physical frailty [5, 6] and cognitive impairment and dementia [7]. Between 25-50% of those over 85 years are estimated to be frail [8], placing them at increased risk of death and disability and admission to hospital and long term care [9]. Dementia contributes a bigger disease burden than other long term illness such as cancer or stroke, with considerable care costs, especially in the last year of life [7, 10].

Primary care services are central to the provision of health care in many developed countries, including the UK. Family physicians, or General Practitioners (GPs), and their teams provide the first point of contact for patients, diagnose disease, monitor long term conditions and have a pivotal role in disease prevention. It has long been acknowledged that primary care-led healthcare systems deliver more efficient and equitable services [11], with healthier, more satisfied patients, for lower cost and with fewer inequalities in both health and access to care [12, 13]. With a rapidly ageing population, the resulting larger proportion experiencing multi-morbidity, cognitive decline and frailty, could place considerable pressures on health and social care provision, especially primary and community care services, in a system where the former is the first and main source of health care. However in the UK, primary care services are already almost at 'saturation point' with substantial increases in consultation rates and consultation duration with the population as a whole [14].

The aim of this paper is to describe, using data from the Newcastle 85+ study, the use of primary and secondary care services by a cohort of the very old as they age over a 5 year period.

Methods

The Newcastle 85+ Study is a prospective observational longitudinal study of a 1921 birth cohort who turned 85 during 2006 [2, 3]. Potential participants were recruited from GP registered patient lists in Newcastle upon Tyne and North Tyneside: contact was established

For peer review only - http://bmjopen.bmj.com/site/about/guidelines.xhtml

with 97% (n=1409/1459) of eligible 85 year olds. Consent was obtained from 74% (n=1042/1409); 851 agreed to undergo detailed multidimensional health assessment (MDHA) and a general practice medical records review (GPRR); 3 consented to MDHA only; 188 consented to GPRR only and 358 declined all participation. Analysis of response, attrition and comparison with the national birth cohort have already been published [2, 3].

As part of their GPRR, participant's primary health care use was recorded for the 12 months prior to their assessment interview (baseline, 36 and 60 months). At baseline and 36 months information gathered included consultations with 16 different professionals seen during these periods. Data for each participant was summarised in 3 ways: total number of consultations with each of the professionals separately; total number of consultations with any primary care professional (GP, GP out of hours, practice nurse/practitioner/healthcare assistant (HCA), community nurse, health visitor); and total number of visits to any of the 16 professionals (Table 1). At 60 months only GP and non-GP primary care consultation were identified with remaining professionals (GP out of hours, practice nurse/practitioner/healthcare assistant (HCA), community nurse, health visitor) as at baseline and 36 months (Table 1)

Additional information on secondary care use was collected for all participants at interview: inpatient, day hospital (total number of days spent in the 12 months prior to interview); outpatient, and accidents and emergency (A&E) (total number of visits in the 3 months prior to interview) (Table 1). Sociodemographic and health characteristics of participants were collected at baseline, 36 and 60 months follow up. The study was approved by the Newcastle and North Tyneside 1 Research Ethics Committee (reference number 06/Q0905/2).

Statistical Analysis

Baseline sociodemographic (living status; self-rated health; education) and health characteristics (Mini-Mental State Examination (MMSE); Geriatric Depression score (GDS); disability; disease group count) of participants and sex differences were analysed using X^2 test for categorical data and Mann-Whitney U for count data. Trends in health care use over time were analysed by negative binomial regression as the data was over dispersed (variance much greater than mean). Zero-inflated negative binomial regression models were used for outcomes where there was high numbers of zero consultations. Final models were adjusted for sex, sociodemographic and health characteristics. Confounding factors (living status, self-rated health, MMSE, GDS, disability and disease count) were measured at multiple time points (apart from education) and values were updated in models. Time trends were reported

For peer review only - http://bmjopen.bmj.com/site/about/guidelines.xhtml

as incidence rate ratios (IRR). Primary and secondary care usage were analysed in the overall sample and in participants who took part at all three time points (baseline, 36, 60 months). All analyses were undertaken in Stata 12.0 (StataCorp; College Station, TX, USA).

Results

At baseline the study comprised 845 participants (319 men and 526 women) of whom 10.2% (n=86) were living in residential care, 12.5% (n=105) had moderate or severe cognitive impairment (MMSE score 18 or less), 6.3% (n=53) had severe disability and 18.6% (n=157) had four or more diseases (Table 2).

Between ages 85 and 90 years, the mean number of all consultations increased significantly by 2.9 extra consultations ($p<0.001$) and the mean number of GP consultations by 1.6 ($p<0.001$) (Table 3). There was an increase in primary care consultations of 0.8 consultations between ages 85 and 88 of which the majority (0.6 consultations) were with the GP (Table 3). The same pattern of consultation use over time was found when the analysis was confined to participants who were alive at all three time points (Table 3). After adjustment for confounding factors there was a significant increase over the five years in all consultations (IRR=1.03, 95%CI 1.01 to 1.05, p=0.001) and GP consultations (IRR=1.03, 95%CI 1.01 to 1.05, p=0.006) (Figure 1).

Analysis of the change in secondary care use between ages 85 and 90 years revealed a non-significant increase in mean inpatient days of 3.8 days (p=0.071), although when restricted to participants who survived to age 90 the mean inpatient days increased by 5 days (p=0.010) (Table 3). No significant changes in mean number of days as a day patient, outpatient or visits to A&E were found (Table 3). After adjustment for confounding factors, no significant trends over time were found for any of the secondary health care use (inpatient days, day hospital, outpatient visits, A&E visits) (Figure 2). Conclusions remained unchanged when analysis was confined to participants who survived the five years (data not shown).

Discussion

Our study suggests that over the age of 85 years, older people are increasingly likely to consult their GP within the primary care team for their health care needs; indeed, by the age of 90 years, most primary care consultations are with the GP. In contrast, no significant changes were found in the use of secondary care services, including A&E and outpatient

For peer review only - http://bmjopen.bmj.com/site/about/guidelines.xhtml

clinics. These patterns remained after adjustment for changing sociodemographic factors (including admission to care homes and health factors such as multi-morbidity and declining cognitive function). These findings help to explain the increasing workload in UK primary care; if GPs are consulting with the growing and increasingly complex population of 85 year olds, who show no increase in use of secondary care services [14].

Strengths and limitations

This study analysed a unique dataset on a large cohort of older adults' use of services. The extraction of data direct from GP medical records is a key strength, as it avoids the potential bias and inaccuracies of data that are self-reported or extracted from research databases. The absence of any information on consultation length and complexity precludes comment on the detailed nature of the increased workload in primary care. One limitation of our data is the less fine-grained coding of professionals consulted at 60 months to reduce data collection time. This meant that increases in consultations by individual primary care professionals could not be compared over the whole five year period between ages 85 and 90. However since the vast majority of the increases in consultations between age 85 and 88 were with the GP, it seems unlikely this trend would be reversed in favour of other professionals. Consultations were analysed for the 12 months prior to each interview therefore excluding data on those who had not been interviewed at that time, mostly due to death. Our estimates of healthcare use are therefore conservative since healthcare use at end of life can be intensive in the 12 months leading to death.

Such findings are of considerable concern for the UK in terms of ensuring both the current, and future, medical workforce is adequately equipped to meet the needs of our ageing population. Strangely, geriatric experience is not a core part of GP training, and clinical teaching in this area within undergraduate medical curricula is limited [15]. It is interesting to note that recent national recommendations to extend core GP training in the UK from 3 years to 4 years, with a focus on the management of age-related issues such as multi-morbidity, frailty and cognitive impairment and dementia, remain as recommendations and have not been translated into practice [16]. Although GP training and primary healthcare provision varies between countries, ageing is a global issue and there are already concerns that current specialist-led models of care provision are not sustainable to meet future demand [17]. Thus whilst increased geriatric training for GPs may help, other issues inherent within health care systems need to be addressed such as the location of specialist geriatric teams, which may be

For peer review only - http://bmjopen.bmj.com/site/about/guidelines.xhtml

more appropriately placed within community care rather than hospital services, and how GPs are rewarded or reimbursed for providing such complex and challenging care [18].

In a majority of high income countries, general or family practice is the mainstay of health care, providing first line contacts and acting as gatekeeper to secondary care [19]. Our findings add further weight to the growing concern that the National Health Service (NHS) primary care will struggle to meet the needs of a rapidly ageing population, in the face of declining GP recruitment [20, 21]. Recent research, looking at over 100 million primary care consultations for all age groups between 2007 and 2014, found that GP workload rose by more than 16% compared to <1% for practice nurses[14]; consultations rates were highest for the very young (< 4 years) and the very old (85 years). The authors concluded such an increase was probably an under-estimate, as the data excluded other GP duties such as administration and teaching. They also found that GP consultations were becoming longer. In England, an average GP consultation is 10 minutes, but longer for people aged over 65 years [22]. For people aged 85 and over where there are high rates of sensory impairment [3 5] and multi-morbidity is the norm, such consultations may be longer and more complex. The skills required may explain the importance of the GP as healthcare provider to this population, despite the rapidly increasing role of nurses and nurse practitioners in primary care [18].

The number of nursing and residential home is decreasing [23], while the number of older people with significant care needs living at home is increasing [24]. This combination can only increase the pressure on primary and community care services [18, 22], while continued financial austerity requires increased cost efficiency in service provision. Better access to geriatric expertise, through community-based multi-disciplinary assessment teams, may in future be beneficial to both patients and our primary gatekeeper healthcare services by providing the latter with easier access to specialist knowledge and support [18, 22]. Although our findings currently reveal the GP as the key care provider for the very old, the crisis in recruitment of doctors suggest that the potential of specialist nurse practitioners to improve patient and care outcomes should be considered. Whether such a service would be acceptable to older people as an alternative to seeing the GP requires further exploration, but the integration of specialist palliative care nurses into routine NHS care provide an encouraging precedent [25, 26].

In summary if GPs are the central care provider for our older people, they must be knowledgeable, skilled, and better supported by appropriately located specialist services to ensure our medical workforce is equipped to meet the needs and demands of a 21st century

For peer review only - http://bmjopen.bmj.com/site/about/guidelines.xhtml

ageing population. In addition to the inclusion of geriatrics in GP training, the provision of such teaching within medical undergraduate curricula needs to be urgently reviewed, in terms of the nature, content and timing of such teaching, in order that future generations of doctors, not just GPs, are adequately prepared. Finally future research is required to explore how best to configure services to address the health care needs of older people whilst maintaining quality of care; such studies must include the very old, a subgroup often neglected from research trials, to ensure their future care is truly evidence based [27].

For peer review only

References

1 Office for National Statistics (ONS). National Population Projections: 2014-based Statistical Bulletin; 2015 29/10/2015.

2 Davies K, Kingston A, Robinson L, Hughes J, Hunt JM, Barker SA, et al. Improving retention of very old participants in longitudinal research: experiences from the Newcastle 85+ study. PloS one. 2014;9(10):e108370.

3 Collerton J, Davies K, Jagger C, Kingston A, Bond J, Eccles MP, et al. Health and disease in 85 year olds: baseline findings from the Newcastle 85+ cohort study. Bmj. 2009 Dec 22;339:b4904.

4 Jagger C, Collerton JC, Davies K, Kingston A, Robinson LA, Eccles MP, et al. Capability and dependency in the Newcastle 85+ cohort study. Projections of future care needs. BMC geriatrics. 2011 May 04;11:21.

5 Clegg A, Young J, Iliffe S, Rikkert MO, Rockwood K. Frailty in elderly people. Lancet. 2013 Mar 02;381(9868):752-62.

6 Rockwood K, Song X, MacKnight C, Bergman H, Hogan DB, McDowell I, et al. A global clinical measure of fitness and frailty in elderly people. CMAJ : Canadian Medical Association journal = journal de l'Association medicale canadienne. 2005 Aug 30;173(5):489-95.

7 Alzheimer's Disease International. World Alzheimer Report 2015. London; 2015 August 2015.

8 Fried LP, Tangen CM, Walston J, Newman AB, Hirsch C, Gottdiener J, et al. Frailty in older adults: evidence for a phenotype. The journals of gerontology Series A, Biological sciences and medical sciences. 2001 Mar;56(3):M146-56.

9 Song X, Mitnitski A, Rockwood K. Prevalence and 10-year outcomes of frailty in older adults in relation to deficit accumulation. Journal of the American Geriatrics Society. 2010 Apr;58(4):681-7.

10 Alzheimer's Society. Dementia UK: Update. London; 2014 November 2014.

11 Starfield B, Shi L, Macinko J. Contribution of primary care to health systems and health. The Milbank quarterly. 2005;83(3):457-502.

12 Starfield B. The future of primary care: refocusing the system. The New England journal of medicine. 2008 Nov 13;359(20):2087, 91.

13 Starfield B, Shi L, Grover A, Macinko J. The effects of specialist supply on populations' health: assessing the evidence. Health affairs. 2005 Jan-Jun;Suppl Web Exclusives:W5-97-W5-107.

14 Hobbs FD, Bankhead C, Mukhtar T, Stevens S, Perera-Salazar R, Holt T, et al. Clinical workload in UK primary care: a retrospective analysis of 100 million consultations in England, 2007-14. Lancet. 2016 Jun 04;387(10035):2323-30.

15 Tullo ES, Spencer J, Allan L. Systematic review: helping the young to understand the old. Teaching interventions in geriatrics to improve the knowledge, skills, and attitudes of undergraduate medical students. Journal of the American Geriatrics Society. 2010 Oct;58(10):1987-93.

16 Gerada CR, B.; Simon, C.;. Preparing The Future GP: The Case for Enhanced GP Training: Royal College of Physicians, RCGP; 2012 April 2012.

17 Alzheimer's Disease International. World Alzheimer Report 2016: Improving healthcare for people living with dementia. London; 2016 September 2016.

18 Robinson L. Present and future configuration of health and social care services to enhance robustness in older age. London 2015.

19 Starfield B, Shi L, Grover A, Macinko J. The effects of specialist supply on populations' health: Assessing the evidence. Health affairs. 2005 May-Jun;24(3):W97-W107.

20 Roland M, Everington S. Tackling the crisis in general practice. Bmj. 2016 Feb 17;352:i942.

21 Thompson M, Walter F. Increases in general practice workload in England. Lancet. 2016 Jun 04;387(10035):2270-2.

For peer review only

For peer review only - http://bmjopen.bmj.com/site/about/guidelines.xhtml

22 Royal College of General Practitioners (RCGP). The 2022 GP: Compendium of evidence; 2013 20th May 2013.
23 Matthews FE, Bennett H, Wittenberg R, Jagger C, Dening T, Brayne C, et al. Who Lives Where and Does It Matter? Changes in the Health Profiles of Older People Living in Long Term Care and the Community over Two Decades in a High Income Country. PloS one. 2016;11(9):e0161705.
24 Jagger C. Educational Disparities in Adult Disability: Person, Place, Policies, and Family. American journal of public health. 2017 Jul;107(7):1021-2.
25 Beswick AD, Rees K, Dieppe P, Ayis S, Gooberman-Hill R, Horwood J, et al. Complex interventions to improve physical function and maintain independent living in elderly people: a systematic review and meta-analysis. Lancet. 2008 Mar 01;371(9614):725-35.
26 Low LF, Yap M, Brodaty H. A systematic review of different models of home and community care services for older persons. BMC health services research. 2011 May 09;11:93.
27 Arthur A, Jagger C. Clinical Trials involving Older People. In: Machin D, Day S, Green S, eds. *Textbook of Clinical Trials*. Second ed. Chichester: Wiley 2006.

For peer review only

For peer review only - http://bmjopen.bmj.com/site/about/guidelines.xhtml

Table 1: Description of outcomes and confounding factors included

Variable	Variable Description	Variable Type
**Primary health care use**		
GP Practice		
GP Practice out of hours		
Practice Nurse/Practitioner/HCA		
Community Nurse		
Health Visitor		
Dietician	This variable records all consultations participants had with a health care professional 12 months prior to each MDHA at each time point.	Outcome
Phlebotomist		
Other		
Not Specified		
Clerical		
Pharmacist/Pharmacy Technician		
Chiropodist/Podiatrist		
Physiotherapist		
Counsellor/Practice Counsellor		
Psychiatrist		
Mental Health Worker		
**Secondary health care use**	Time spent by participants for each different type of hospital admission.	Outcome
Inpatient	Days spent during the 12 month prior to MDHA	
Day Patient		
Outpatient	Number of visits during the 3 months prior to MDHA	
A&E		
**Time**	This is a continuous measure of time in years from the start of baseline interview to participant's death.	Covariate
**Living status***		
Alone in community	Participant's living arrangements at each MDHA	Covariate
Not alone in community		
Institutional living		
**Self-Rated Health**	Participant's perception of their	Covariate

For peer review only - http://bmjopen.bmj.com/site/about/guidelines.xhtml

	Excellent/Very Good<br>Good<br>Fair/Poor	general health on a five point scale recoded into 3 categories.	
**MMSE***			
	Normal (26-30)<br>Mild (22-25)<br>Mod (18-21)<br>Severe (0-17)	Participant's categorised MMSE scores at each MDHA.	Covariate
**GDS***			
	No depression<br>Mild<br>Severe<br>MMSE<15	Categorised Geriatric depression score collected at each MDHA.	Covariate
**Categorised Disability***			
	None<br>1 - 6<br>7 - 12<br>13 - 17	Categorised disability score based on activities of daily living (ADLs), collected at each MDHA.	Covariate
**Disease Groups***	0<br>1<br>2 – 3<br>4+	Categorised disease groups (max 8). 8 Disease groups were identified with each scored 1 if the Participant's had a GP diagnoses of said disease at each GPRR. Disease groups included: Arthritis, Cancer, Cardiac disease, Cerebrovascular disease, Diabetes mellitus, Hypertension, Respiratory disease and Cognitive Impairment.	Covariate

MDHA (Multidimensional Health Assessment); GPRR (GP Record Review); HCA (Healthcare Assistant); A&E (Accidents and Emergency); MMSE (Mini-Mental Estate Examination); GDS (Geriatric Depression Score);

For peer review only - http://bmjopen.bmj.com/site/about/guidelines.xhtml

Table 2: Baseline sociodemographic and health characteristics of the 85+ study participants

Characteristic[1]	Males (319)	Females (526) %(N)	All (845)	P-value
**Living Status**				
Alone in community	39.5 (126)	64.0 (336)	54.7 (462)	
Not alone in community	54.2 (173)	23.4 (123)	35.1 (296)	<0.001
Institutional living	6.3 (20)	12.6 (66)	10.2 (86)	
**Self-rated health**				
Excellent/Very Good	43.9 (137)	37.7 (193)	40.1 (330)	
Good	36.5 (114)	38.3 (196)	37.6 (310)	0.152
Fair/Poor	19.6 (61)	24.0 (123)	22.3 (184)	
**Education**				
0-9 Years	62.3 (195)	65.7 (339)	64.4 (534)	
10-11 Years	24.6 (77)	21.7 (112)	22.8 (189)	0.576
12+ Years	13.1 (41)	12.6 (65)	12.8 (106)	
**MMSE[2]**				
Normal (26-30)	71.9 (228)	71.1 (371)	71.4 (599)	
Mild (22-25)	18.3 (58)	14.8 (77)	16.1 (135)	0.113
Mod (18-21)	3.5 (11)	6.9 (36)	5.6 (47)	
Severe (0-17)	6.3 (20)	7.3 (38)	6.9 (58)	
**GDS[3]**				
No depression	79.7 (247)	71.4 (360)	74.6 (607)	
Mild	9.0 (28)	13.9 (70)	12.0 (98)	0.066
Severe	6.8 (21)	8.5 (43)	7.9 (64)	
MMSE<15	4.5 (14)	6.2 (31)	5.5 (45)	
**Categorised Disability**				
None	31.6 (100)	16.3 (85)	22.1 (185)	
1 - 6	52.4 (166)	57.5 (300)	55.5 (466)	<0.001
7 - 12	11.7 (37)	18.8 (98)	16.1 (135)	
13 - 17	4.4 (14)	7.5 (39)	6.3 (53)	
**Disease Groups[4]**				
0	6.6 (21)	4.2 (22)	5.1 (43)	
1	19.4 (62)	21.5 (113)	20.7 (175)	0.448
2 – 3	55.5 (177)	55.7 (293)	55.6 (470)	
4+	18.5 (59)	18.6 (98)	18.6 (157)	

[1]Data available at each time point for all characteristics except education;
[2]MMSE (Mini-Mental Estate Examination); [3]GDS (Geriatric Depression Score);
[4]For diseases included see Table 1

For peer review only

Table 3: Mean number of consultations (healthcare use) at each time point of the study for all participants, by sex

**All Participants (N=845)**	Baseline (N=845)	36 Months (N=485) Mean (SD)	60 Months (N=344)	P-value
All Consultations	10.4 (7.7)	11.4 (8.3)	13.3 (13.6)	<0.001
Primary Care Consultations	9.8 (7.5)	10.6 (7.8)	- [1]	0.064
GP	5.9 (4.8)	6.5 (5.9)	7.5 (6.5)	<0.001
GP out of hours service	0.1 (0.5)	0.2 (0.8)	- [1]	0.575
Practice Nurse/Practitioner/HCA**	2.8 (3.0)	2.6 (3.0)	- [1]	0.634
Community Nurse**	1.0 (3.9)	1.1 (3.0)	- [1]	0.823
Clerical	0.3 (0.7)	0.3 (1.6)	5.8 (10.7)	<0.001
Pharmacist/Pharmacy Technician	0.1 (0.3)	0.0 (0.0)	0.0 (0.3)	0.693
All Other Consults	0.2 (0.7)	0.5 (1.1)	0.0 (0.5)	<0.001
Inpatient	3.6 (15.3)	4.6 (14.0)	7.4 (18.6)	0.071
Day Patient	0.2 (0.9)	0.2 (0.6)	0.1 (0.4)	0.027
Outpatient[2]	0.6 (1.8)	0.6 (1.2)	0.6 (1.9)	0.974
A & E[2]	0.1 (0.3)	0.1 (0.4)	0.1 (0.4)	0.500
**Participants Alive at 60 Months (N=344)**	**(N=344)**	**(N=344)**	**(N=344)**	
All Consultations	9.9 (6.6)	10.8 (8.1)	13.3 (13.6)	<0.001
Primary Care Consultations	9.4 (6.5)	10.0 (7.5)	- [1]	0.281
GP	5.7 (4.5)	6.2 (6.0)	7.5 (6.5)	<0.001
GP out of hours service**	0.1 (0.3)	0.2 (0.9)	- [1]	0.118
Practice Nurse/Practitioner/HCA**	3.2 (3.3)	2.8 (2.9)	- [1]	0.161
Community Nurse**	0.5 (2.0)	0.8 (2.0)	- [1]	0.473
Clerical	0.3 (0.7)	0.4 (1.8)	5.8 (10.7)	<0.001
Pharmacist/Pharmacy Technician	0.1 (0.3)	0.0 (0.2)	0.0 (0.3)	0.448
All Other Consults	0.2 (0.6)	0.4 (1.1)	0.0 (0.1)	<0.001
Inpatient	2.4 (9.9)	3.5 (11.5)	7.4 (18.6)	0.010
Day Patient	0.2 (0.7)	0.2 (0.6)	0.1 (0.4)	0.373
Outpatient [2]	0.5 (1.0)	0.5 (1.2)	0.6 (1.9)	0.069
A&E[2]	0.1 (0.3)	0.1 (0.4)	0.1 (0.4)	0.896

*p-value for change over time
[1]Not available at 60 months[2] Numbers based on 3 months prior to interview

HCA (Healthcare Assistant); A&E (Accidents and Emergency)

For peer review only - http://bmjopen.bmj.com/site/about/guidelines.xhtml

Figure 1: Time trends in primary and community care consultations (IRR and 95% CI) adjusted for sex, living status, self-rated health, MMSE (Mini-Mental Estate Examination), GDS (geriatric depression score) and disease groups count. Primary care, practice nurse\practitioner and community nurse analysed between baseline and 36 months.

Figure 2: Time trends in secondary care consultations (IRR and 95% CI) adjusted for sex, living status, self-rated health, MMSE (Mini-Mental Estate Examination), GDS (geriatric depression score) and disease groups count. A&E (Accidents and Emergency).

Contributor statement

LR conceived the study, obtained project funding and drafting of the paper. MEY, CJ and TF were responsible for data analysis and drafting of the paper. RD, BH and SP contributed to drafting of the paper. All authors approved the final manuscript.

Funding

UK MRC and BBSRC (G0500997), Dunhill Medical Trust (R124/0509); Newcastle Healthcare Charity; NIHR Newcastle Biomedical Research Centre. The funding sources had no role in the study design; in the collection, analysis and interpretation of data; in the writing of the report, or in the decision to submit the paper for publication. This paper presents independent research funded by the National Institute for Health Research School for Primary Care Research (NIHR SPCR), project number SPCR 303. Louise Robinson is funded by a National Institute for Health Research Professorship (NIHR-RP-011-043). The views expressed are those of the author(s) and not necessarily those of the NIHR, the National Health Service or the Department of Health.

Acknowledgements

Thanks are especially due to the 85 year olds of Newcastle and North Tyneside, and their families and carers, for the generous donation of their time and personal information. In addition we thank the research nurses, data manager, project secretary, and the North of England Commissioning Support Unit and local general practitioners and their staff.

Copyright

The Corresponding Author has the right to grant on behalf of all authors and does grant on behalf of all authors, a worldwide licence to the Publishers and its licensees in perpetuity, in all forms, formats and media (whether known now or created in the future), to i) publish, reproduce, distribute, display and store the Contribution, ii) translate the Contribution into

For peer review only - http://bmjopen.bmj.com/site/about/guidelines.xhtml

other languages, create adaptations, reprints, include within collections and create summaries, extracts and/or, abstracts of the Contribution, iii) create any other derivative work(s) based on the Contribution, iv) to exploit all subsidiary rights in the Contribution, v) the inclusion of electronic links from the Contribution to third party material where-ever it may be located; and, vi) licence any third party to do any or all of the above.

Competing interest statement

All authors have completed the Unified Competing Interest form and declared any relevant support from any organisation for the submitted work in the previous three years.

Transparency declaration

LR affirms that the manuscript is an honest, accurate, and transparent account of the study being reported; that no important aspects of the study have been omitted; and that any discrepancies from the study as planned (and, if relevant, registered) have been explained.

Data Sharing Statement

Newcastle 85+ Study data may be obtained by agreement from the Data Guardians Group on submission of a data request form (available at: https://research.ncl.ac.uk/85plus/datarequests).

For peer review only

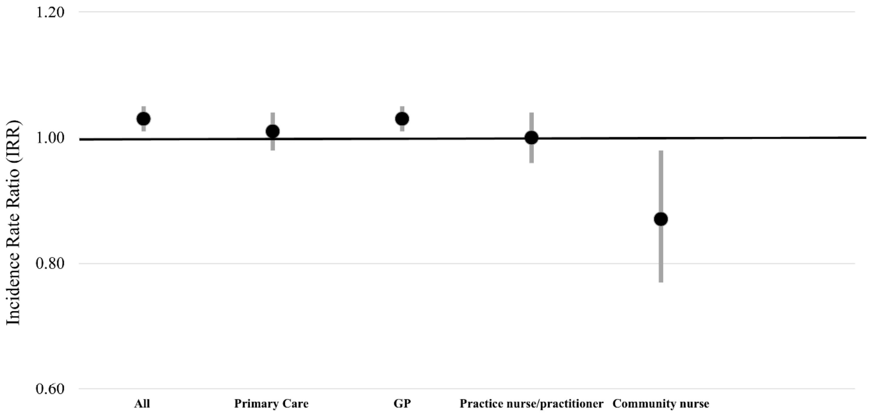

Figure 1: Time trends in primary and community care consultations (IRR and 95% CI) adjusted for sex, living status, self-rated health, MMSE (Mini-Mental Estate Examination), GDS (geriatric depression score) and disease groups count. Primary care, practice nurse\practitioner and community nurse analysed between baseline and 36 months.

338x190mm (300 x 300 DPI)

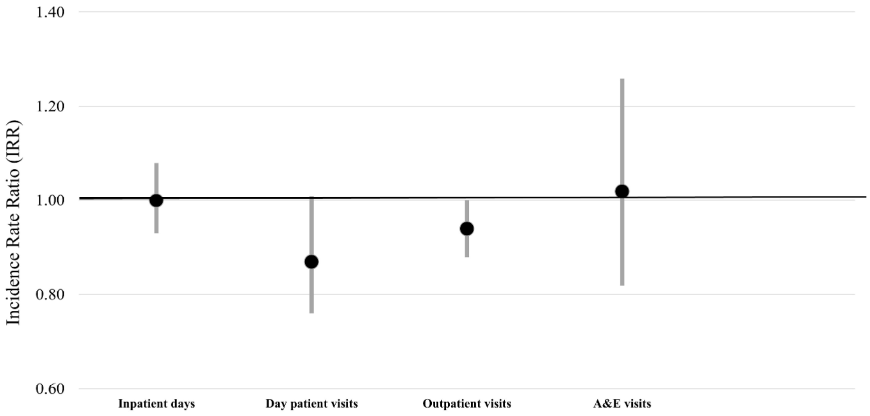

Figure 2: Time trends in secondary care consultations (IRR and 95% CI) adjusted for sex, living status, self-rated health, MMSE (Mini-Mental Estate Examination), GDS (geriatric depression score) and disease groups count. A&E (Accidents and Emergency).

338x190mm (300 x 300 DPI)

STROBE 2007 (v4) Statement—Checklist of items that should be included in reports of *cohort studies*

Section/Topic	Item #	Recommendation	Reported on page #
**Title and abstract**	1	(*a*) Indicate the study's design with a commonly used term in the title or the abstract	1 and 3
		(*b*) Provide in the abstract an informative and balanced summary of what was done and what was found	3
**Introduction**			
Background/rationale	2	Explain the scientific background and rationale for the investigation being reported	5
Objectives	3	State specific objectives, including any prespecified hypotheses	5
**Methods**			
Study design	4	Present key elements of study design early in the paper	5/6
Setting	5	Describe the setting, locations, and relevant dates, including periods of recruitment, exposure, follow-up, and data collection	5/6
Participants	6	(*a*) Give the eligibility criteria, and the sources and methods of selection of participants. Describe methods of follow-up	5/6
		(*b*) For matched studies, give matching criteria and number of exposed and unexposed	-
Variables	7	Clearly define all outcomes, exposures, predictors, potential confounders, and effect modifiers. Give diagnostic criteria, if applicable	6
Data sources/ measurement	8*	For each variable of interest, give sources of data and details of methods of assessment (measurement). Describe comparability of assessment methods if there is more than one group	*6*
Bias	9	Describe any efforts to address potential sources of bias	6
Study size	10	Explain how the study size was arrived at	6
Quantitative variables	11	Explain how quantitative variables were handled in the analyses. If applicable, describe which groupings were chosen and why	6
Statistical methods	12	(*a*) Describe all statistical methods, including those used to control for confounding	6
		(*b*) Describe any methods used to examine subgroups and interactions	6
		(*c*) Explain how missing data were addressed	6
		(*d*) If applicable, explain how loss to follow-up was addressed	6
		(*e*) Describe any sensitivity analyses	6
**Results**			

For peer review only

Participants	13*	(a) Report numbers of individuals at each stage of study—eg numbers potentially eligible, examined for eligibility, confirmed eligible, included in the study, completing follow-up, and analysed	6/7
		(b) Give reasons for non-participation at each stage	7
		(c) Consider use of a flow diagram	-
Descriptive data	14*	(a) Give characteristics of study participants (eg demographic, clinical, social) and information on exposures and potential confounders	7
		(b) Indicate number of participants with missing data for each variable of interest	-
		(c) Summarise follow-up time (eg, average and total amount)	7
Outcome data	15*	Report numbers of outcome events or summary measures over time	7
Main results	16	(*a*) Give unadjusted estimates and, if applicable, confounder-adjusted estimates and their precision (eg, 95% confidence interval). Make clear which confounders were adjusted for and why they were included	7
		(*b*) Report category boundaries when continuous variables were categorized	7
		(*c*) If relevant, consider translating estimates of relative risk into absolute risk for a meaningful time period	7
Other analyses	17	Report other analyses done—eg analyses of subgroups and interactions, and sensitivity analyses	7
**Discussion**			
Key results	18	Summarise key results with reference to study objectives	7
**Limitations**			
Interpretation	20	Give a cautious overall interpretation of results considering objectives, limitations, multiplicity of analyses, results from similar studies, and other relevant evidence	8
Generalisability	21	Discuss the generalisability (external validity) of the study results	8
**Other information**			
Funding	22	Give the source of funding and the role of the funders for the present study and, if applicable, for the original study on which the present article is based	9

*Give information separately for cases and controls in case-control studies and, if applicable, for exposed and unexposed groups in cohort and cross-sectional studies.

Note: An Explanation and Elaboration article discusses each checklist item and gives methodological background and published examples of transparent reporting. The STROBE checklist is best used in conjunction with this article (freely available on the Web sites of PLoS Medicine at http://www.plosmedicine.org/, Annals of Internal Medicine at http://www.annals.org/, and Epidemiology at http://www.epidem.com/). Information on the STROBE Initiative is available at www.strobe-statement.org.

For peer review only
